# Preparation of mesophase pitch by aromatics-rich distillate of naphthenic vacuum gas oil

**DOI:** 10.1007/s13203-015-0123-0

**Published:** 2015-07-09

**Authors:** Ming Li, Dong Liu, Hui Du, Qinyin Li, Xulian Hou, Jiashun Ye

**Affiliations:** 1 State Key Laboratory of Heavy Oil Processing, China University of Petroleum, Qingdao, 266580 China; 2 College of Science, China University of Petroleum, Qingdao, 266580 China; 3 China Petroleum Engineering Co., Ltd., Beijing Company, Beijing, 100085 China

**Keywords:** Mesophase pitch, Naphthenic vacuum gas oil, Optical texture, Molecular structure, Crystal structure

## Abstract

Two aromatics-rich distillates R1 and R2 with different properties from naphthenic base vacuum gas oil were used for preparing mesophase pitch through high-pressure thermal treatment. ^1^H-NMR, FT-IR and VPO were employed to characterize the structural parameters of the raw materials. The products’ optical texture and molecular structure were analyzed by polarized light optical microscopy, ^1^H-NMR, FT-IR and XRD. The effect of raw materials’ structure on the formation of mesophase pitch was discussed. The results showed that the structure of the raw material had an important effect on the formation of mesophase pitch. The raw material R2 with higher aromaticity, more naphthenic structure and less alkyl side chains was easy to form mesophase pitch with large-domains optical texture, lower softening point and more ordered crystal structure.

## Introduction

Mesophase pitch prepared by bitumen, heavy oil, coal tar and other raw materials through thermal condensation reaction is recognized as the precursor of carbon fiber, needle coke, carbon electrode material, foam materials and other raw materials with excellent advanced features [1–4]. It has been well established that the molecular weight and alkyl groups of a raw material are primary factors controlling the solubility and anisotropic of resultant carbons [5–7]. In particular, the high concentration of alkyl group in pitch seemed to increase the solubility of the mesophase, thereby the optical structure of mesophase pitch was improved [8].

Mochida et al. [9–11] reported that hydrogenation and reductive alkylation methods were effective methods to convert the quinolone-insoluble fractions into graphitizable carbon. After studying the preparation of naphthalene mesophase pitch and methyl naphthalene mesophase pitch, Korai and Mochida [12] suggested that the methyl in mesophase molecule was the main factor to the stacking of mesogen molecules. It has been reported that a nematic liquid crystal which was a typical mesophase material was produced by heavy aromatic hydrocarbons during the heat treatment [13, 14]. The heavy fraction of light diesel oil, derived from petroleum naphtha catalytic cracking, was chosen as the additives in co-carbonization of medium coal tar pitch to modify the carbonization property. The results showed that the mesophase pitch optical structure changed from the original mosaic into a large-flow domain [15, 16]. The composition of aromatics-rich fraction in catalytic slurry (FCCRF) and the carbonization behavior have been studied [17]. The result showed that the carbonization behavior was influenced by the composition of feedstock substantially, so the selection of raw materials was very important. To be specific, a mesophase pitch with an orderly disk-like structure could be formed from a feedstock with feasible composition. But the influences of the alkyl side chains and naphthenic groups on the optical and molecular structures of mesophase pitch have not been studied systematically so far as we know.

In this work, two batches of aromatics-rich distillates R1 and R2 with different properties were derived from naphthenic base vacuum gas oil by furfural extraction, then mesophase pitches were prepared by thermal treatment using R1 and R2 as raw materials, respectively, namely R1-MP and R2-MP. The influences of the raw materials on the properties of mesophase pitch were studied, and the carbonization mechanism was discussed preliminarily.

## Experimental

### Materials

Two aromatics-rich distillates R1 and R2 with different properties are derived from naphthenic vacuum gas oil.

The characteristics of R1 and R2 are listed in Table 1. From Table 1, the two oils contain no asphaltene. Compared with R1, R2 contains more aromatics but less saturates and resin. The relative molecular mass of R2 is higher than R1, while the n(H):n(C) was opposite. It indicates that the condensation degree of R2 was higher than that of R1.

**Table 1:** Basic properties of feedstocks

Sample	wt%				wt%		n(H):n(C)	M
	Saturates	Aromatics	Resin	Asphaltene	C	H		
R1	22.26	62.94	13.69	0	87.93	9.94	1.36	383.20
R2	16.51	71.67	10.87	0	90.12	7.49	1.00	526.51

*M* relative molecular mass

### Experimental methods

The raw material was placed in the 100-ml high-pressure autoclave and heated in an electric furnace to 440–450 °C with a rate of less than 3 °C/min after purging the reaction system by nitrogen for three times. During the experiment, the pressure was maintained at about 4 MPa. After the pyrogenation process at 440–445 °C, the reaction was carried out under 4 MPa for 2–12 h.

### Analysis

The elemental compositions of samples were obtained from Germany Chnos company Vario EL II elemental analyzer. The relative molecular mass of the raw materials were obtained from German Knauer Company Vapour Pressure Osmometer K-700 molecular weight apparatus. The microstructures were observed and photographed by BX51-P polarizing microscope with camera (made in Germany). FT-IR spectra were obtained on US Digilet Company FT S215 Fourier transform-infrared spectroscopy. Solid samples were dissolved in benzene first and smeared on NaCl single-crystal flakes uniformly, then characterized after the solution was volatilized. ^1^H-NMR spectra were obtained from US Varian Company Unity-200 MHz FT NMR spectrometer. Tetramethylsilane (TMS) was added as an internal standard to samples that were dissolved in CDCl_3_. The operating frequency is 80 MHz, and the scanning width is 2 kHz. The group composition of mesophase pitch was analyzed according to SH/T 0509-92.

## Results and discussion

### FT-IR analysis

**Fig. 1:**
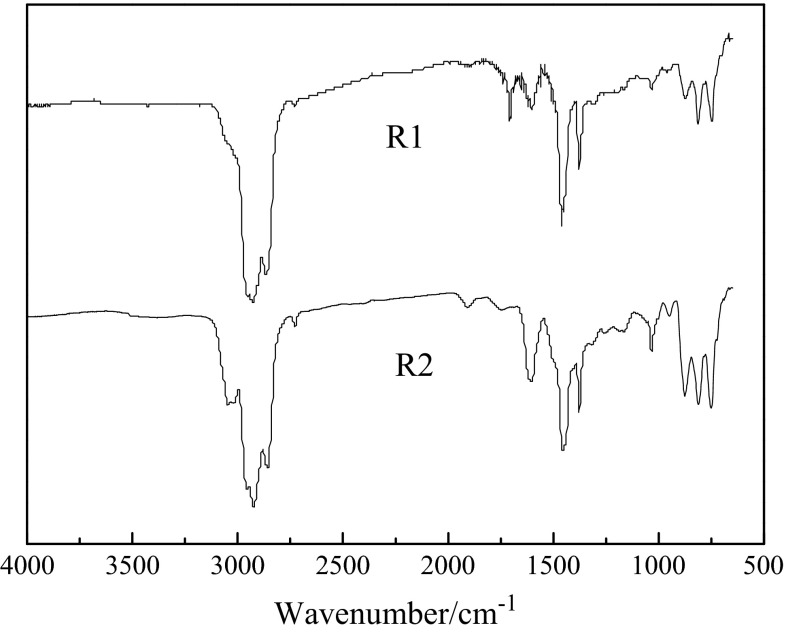
FT-IR spectra of raw materials

The FT-IR spectra of R1 and R2 are shown in Fig. 1. It can be observed from Fig. 1 that two kinds of raw materials present strong absorption peaks at 1460, 2853, 2923, 1380 and 2960 cm^−1^, indicating that the raw materials contain many alkyl side chains. The molar ratio of –CH_2_– (absorption peaks at 1460 cm^−1^) to –CH_3_ (absorption peaks at 1460 cm^−1^) can be evaluated according to the following formula [18].

1 $$ r = 3.07\frac{{A_{1460} }}{{A_{1380} }} - 3.72 $$

In the formula, *A*_1460_ and *A*_1380_ represent absorption peak intensities at 1460 and 1380 cm^−1^. The results showed that the ratios of R1 and R2 were 2.01 and 0.76, suggesting that the alkyl side chain of R2 was shorter than that of R1. The aromaticity (*f*_a_) of R1 and R2 was calculated according to the following formula [18].

2 $$ f_{\text{a}} = 0.574P + 0.024 $$

In the formula, *P* is defined as *A*_1600_/(*A*_1600_ + 0.16 *A*_1460_ + 0.23 *A*_1330_), and *A*_1660_ represents absorption peak intensity at 1660 cm^−1^. The aromaticity (*f*_a_) of R1 and R2 was 0.37 and 0.62, implying that the aromaticity of R2 was higher than that of R1.

**Fig. 2:**
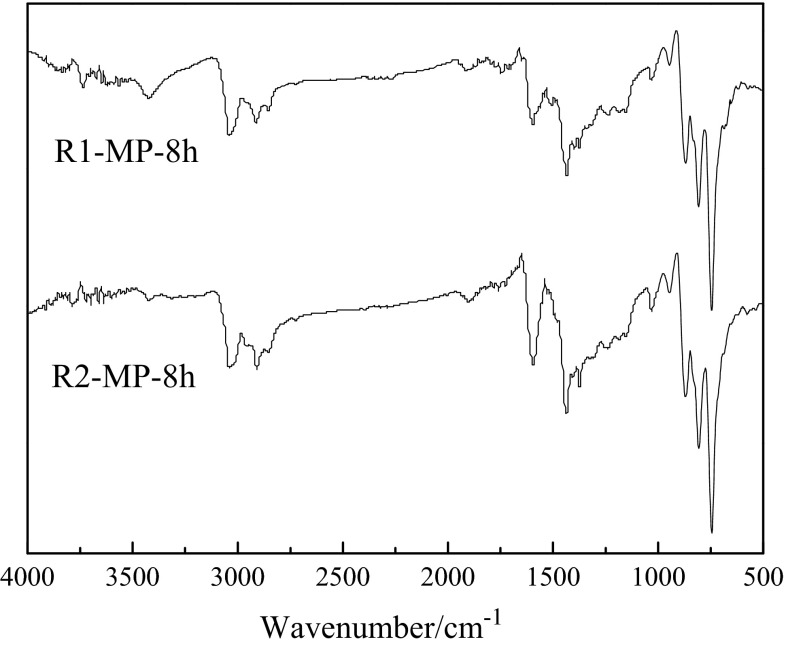
FT-IR spectra of the mesophase pitches

The FT-IR spectra of R1-MP-8h (mesophase pitch prepared from R1 for 8 h) and R2-MP-8h (mesophase pitch prepared from R2 for 8 h) are shown in Fig. 2. The aliphatic hydrocarbon absorption intensity (3000–2845 cm^−1^) of R1-MP-8h and R2-MP-8h increased while condensed aromatics absorption intensity (900–650 cm^−1^, 1650–1450 cm^−1^) decreased as compared to the raw materials. It suggested a fracture process of long aliphatic side chains and a polycondensation process of molecules. The aromatic indexes (*I*_ar_) of R1-MP-8h and R2-MP-8h calculated by the following formula [18] were 0.76 and 0.69, indicating that R2-MP-8h contained more aliphatic groups than R1-MP-8h.

3 $$ I_{\text{ar}} = \frac{{Ab_{3040} }}{{Ab_{3040} + Ab_{2920} }} $$

The 880/1600 cm^−1^ peak intensity ratio (isolated aromatic C–H/aromatic C=C) were used to reflect the condensation degrees of polycyclic aromatic hydrocarbons (PAH) [18], and the PAH of R1-MP-8h and R2-MP-8h were 2.71 and 1.05, demonstrating that the condensation degrees of R2-MP-8h were lower than that of R1-MP-8h. The result was consistent with the change of aromatic indexes.

### ^1^H-NMR analysis

**Fig. 3:**
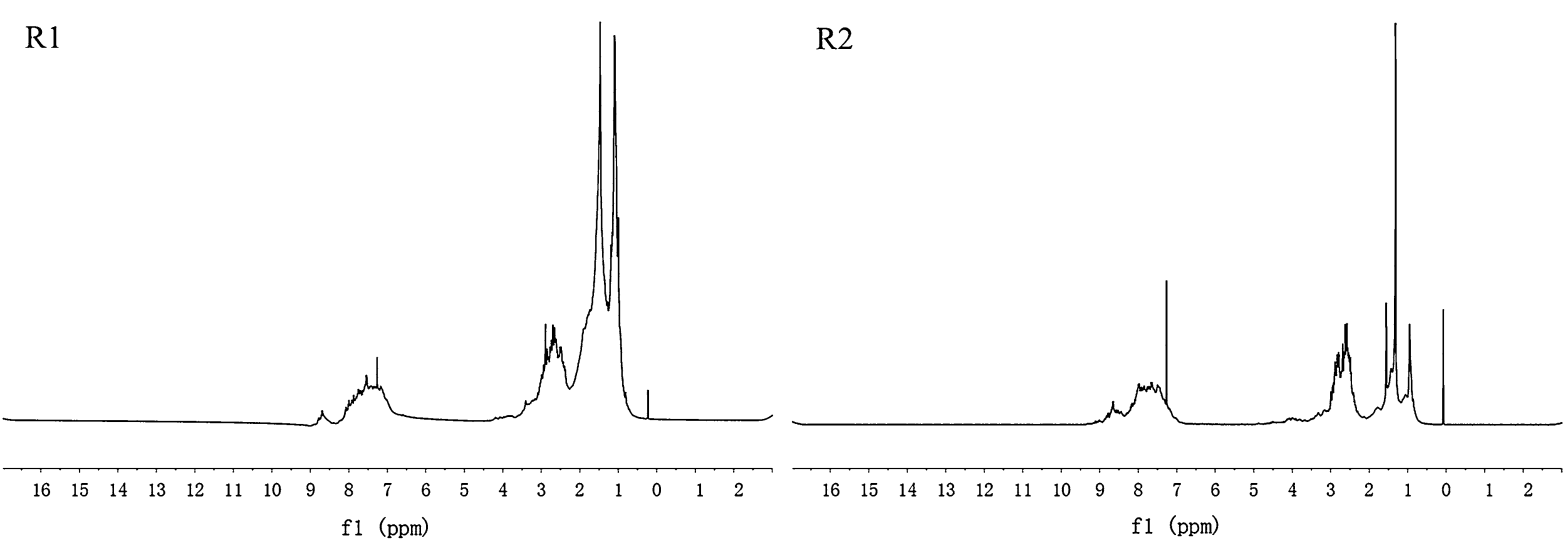
^1^H-NMR spectrum of the raw materials

The ^1^H-NMR spectra of R1 and R2 are shown in Fig. 3 and the analysis results are shown in Table 2. It revealed that the H_α_ and H_γ_ contents of R2 were higher than that of R1, while the H_ar_ content was opposite, indicating that R1 contained more alkyl side chains than R2. The alkyl side chains could improve the reaction rate to a certain extent. But if the raw material contained excessive amounts of alkyl side chains, it would produce more free radicals [19, 20], which make the polycondensation reaction very fast and hinder formation of a mesophase phase with low softening point [9–13]. The H_n_ contents of R1 and R2 were 22.32 and 35.17, illustrating that R2 contained more naphthenic groups. The structural parameters of the raw materials shown in Table 2 were calculated using the improved Brown–Ladner method [18]. The aromaticity (*f*_a_) of R1 and R2 was 0.39 and 0.66, which was consistent with the FT-IR analysis results.

**Table 2:** Structural parameters of the raw materials

Sample	H_ar_	H_α_	H_β_	H_γ_	H_n_	*f*_a_
R1	12.74	21.39	17.53	26.02	22.32	0.39
R2	32.25	14.57	6.49	11.52	35.17	0.66

H_ar_, aromatic hydrogens; H_α_, aliphatic hydrogens in methyl or methylene groups in α-position to an aromatic ring (3.3–2.0 ppm); H_n_, naphthenic hydrogen (2.0–1.4 ppm); H_β_, aliphatic hydrogens in methyl or methylene groups in β-position to an aromatic ring (1.4–1.0 ppm); H_γ_, aliphatic hydrogens in methyl or methylene groups in γ-position to an aromatic ring (1.0–0.5 ppm); *f*_a_, aromaticity

**Fig. 4:**
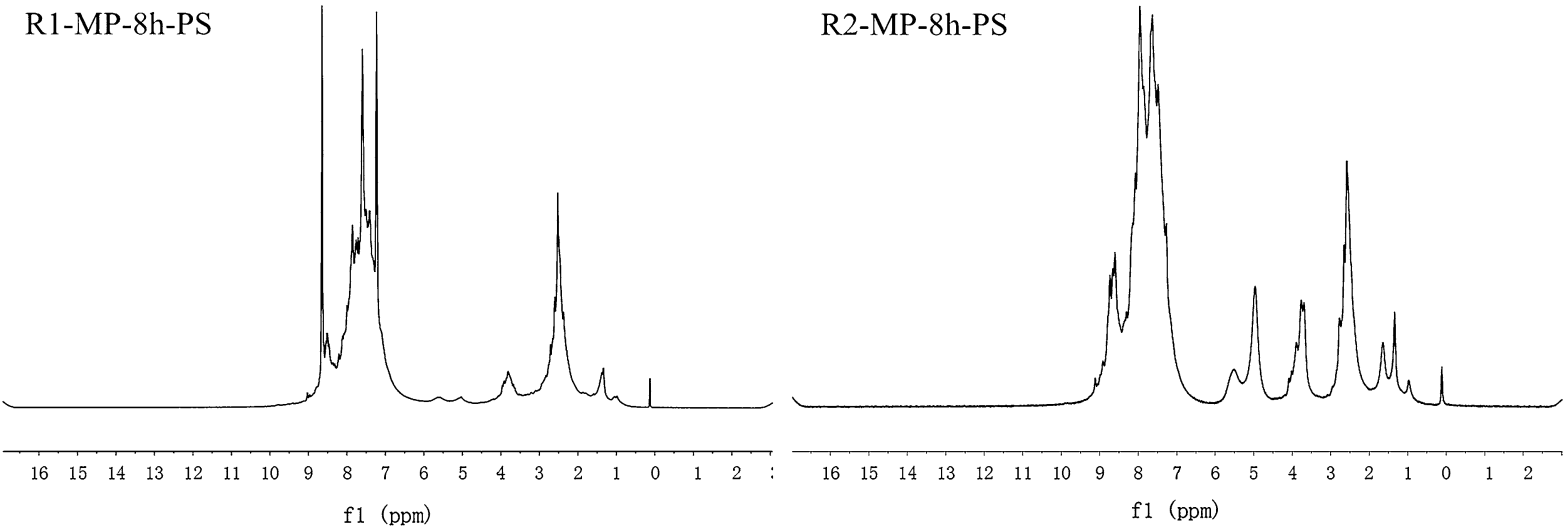
^1^H-NMR spectra of MP-PS

The pyridine-soluble fractions of R1-MP-8h and R2-MP-8h (R1-MP-8h-PS and R2-MP-8h-PS) were analyzed by ^1^H-NMR. The spectra of R1-MP-8h-PS and R2-MP-8h-PS are shown in Fig. 4 and the distribution of the hydrogen atoms is tabulated in Table 3.

**Table 3:** Hydrogen atoms distribution of MP-PS

Samples	H_ar_ (%)	H_α_ (%)	H_β_ (%)	H_γ_ (%)	H_n_ (%)
R1-MP-8h-PS	87.95	5.84	3.81	1.42	0.98
R2-MP-8h-PS	72.71	16.29	4.92	2.36	3.72

As shown in Table 3, the H_α_ and H_γ_ contents of R2-MP-8h-PS were higher than that of R1-MP-8h-PS, while the H_ar_ content was opposite, indicating that R2-MP-8h-PS contained more alkyl side chains than R1-MP-8h-PS. The H_**n**_ content of R1-MP-8h-PS and R2-MP-8h-PS was 0.98 and 3.72, implying that R2-MP-8h-PS contained more naphthenic groups. The absorption peaks of –CH_2_– at 3.3–4.2 indicated that the aromatic layers were connected with each other by –CH_2_– bonds [21]. The contents of H_β_ and H_γ_ in mesophase pitches decreased markedly as compared with the raw materials, illustrating that the pyrolysis process was mainly to remove H_β_ and H_γ_, and their removal resulted in the generation of aromatic intermediates which were stable aromatic radicals [8, 15]. The aromaticity (*f*_a_) of R1-MP-8h-PS and R2-MP-8h-PS calculated by improved n-d-M method [18] was 0.76 and 0.65, implying that R2-MP-8h-PS contained more aliphatic groups, which was consistent to the results of FT-IR analysis.

### Optical textures analysis

**Fig. 5:**
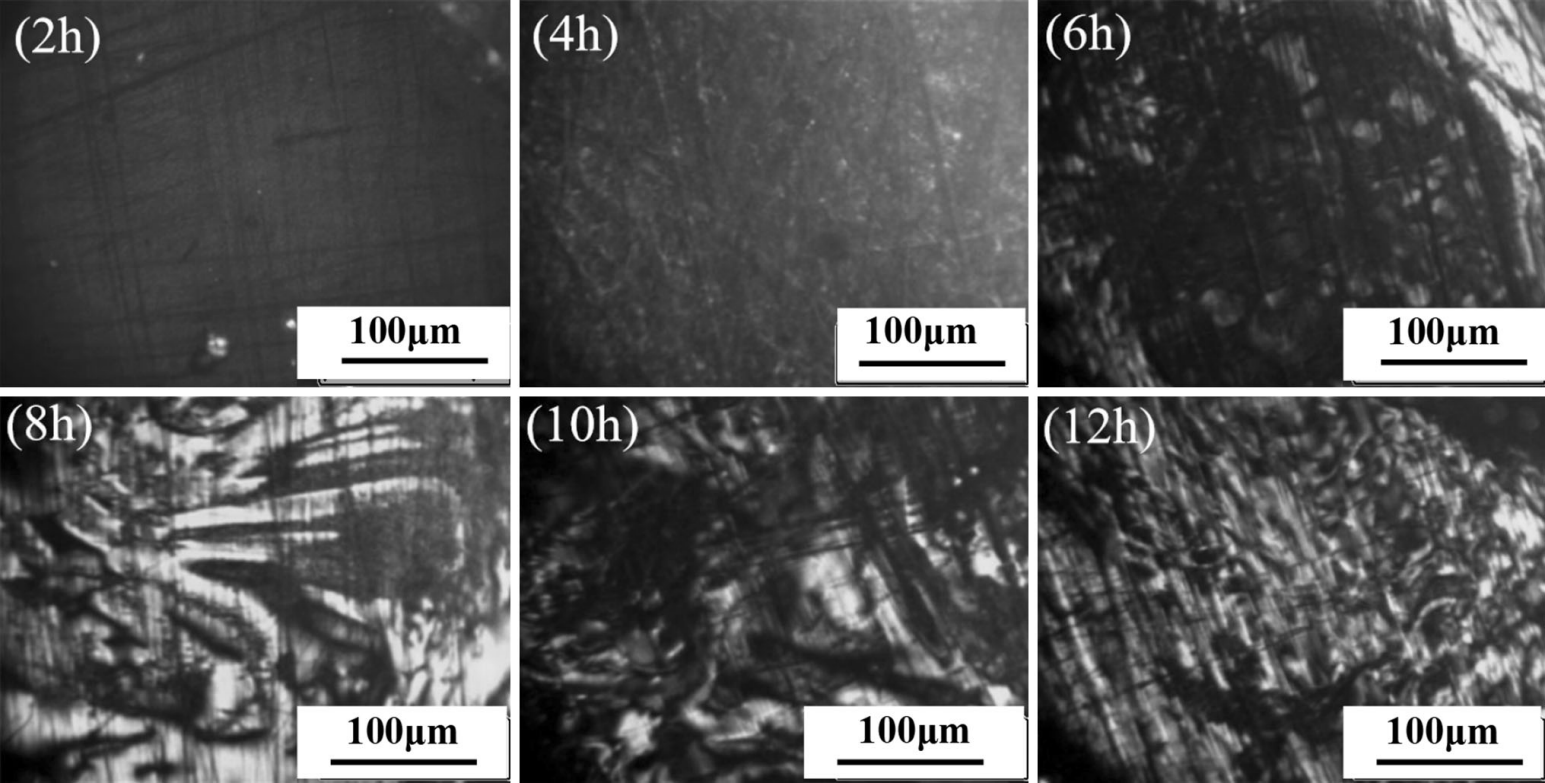
Optical micrographs of R1-MP

**Fig. 6:**
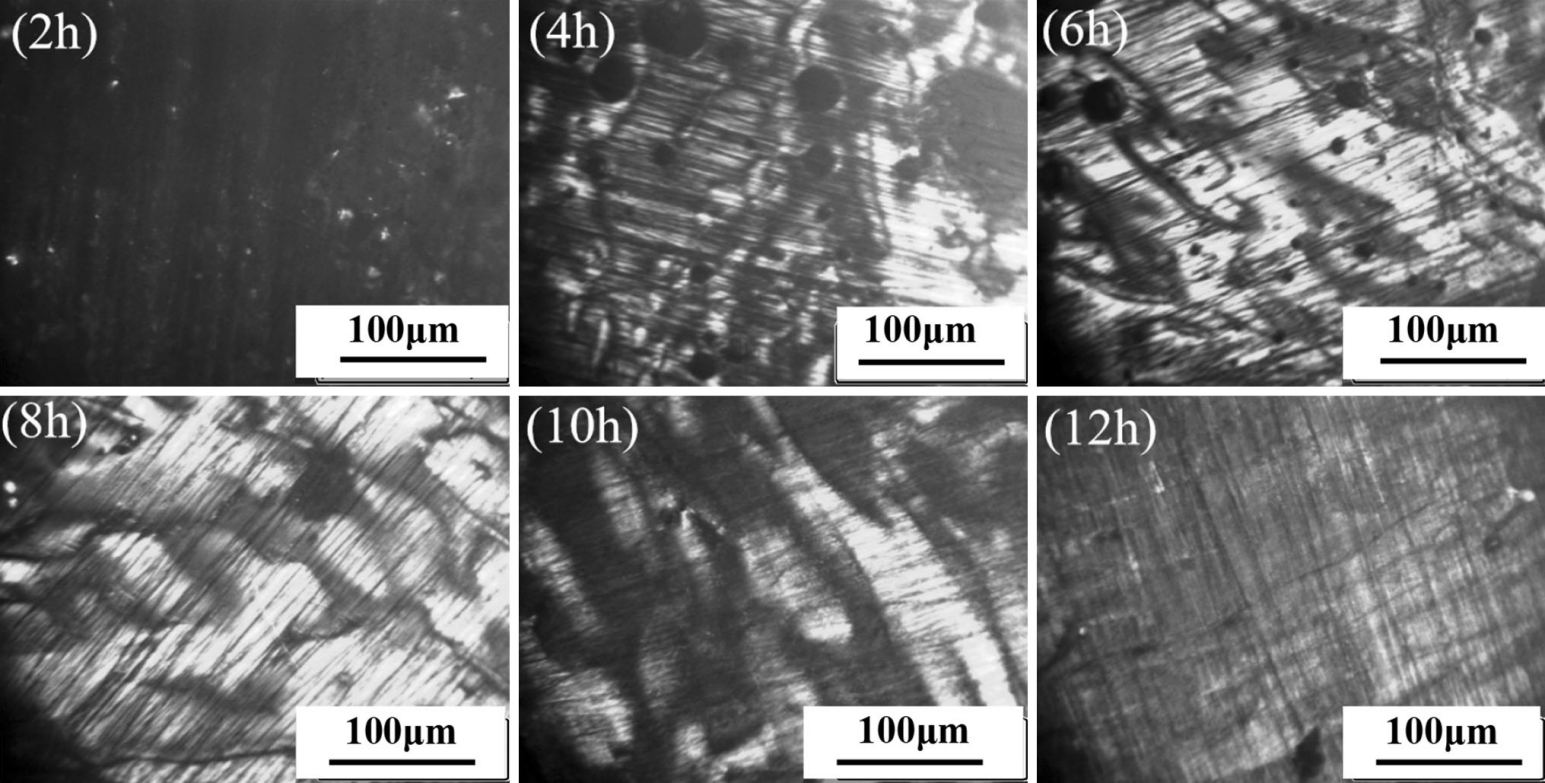
Optical micrographs of R2-MP

The optical micrographs of R1-MP and R2-MP are shown in Figs. 5 and 6. As shown in Fig. 5, a number of small spheres and a few of ball aggregates were formed after 4 h. A mesophase pitch with fine mosaic structure was generated after 6 h. With the development of the reaction, it was observed that the mesophase pitch with medium domain structure (diameter in 100 μm–200 μmon average) was formed after 8 h. Moreover, the mesophase content of R1-MP-8h was 87 % and the softening point was 289 °C. When the carbonization time was too long (over 12 h), a mesophase pitch with fine mosaic structure was formed, because the over-carbonization destroyed the crystal structure. From the optical micrographs of R2-MP shown in Fig. 6, we found that the changing tendency of R2-MP was consistent with that of R1-MP. Due to the high condensation degree of R2, it just needed 4 h to obtain a mesophase pitch with coarse mosaic structure. A mesophase pitch with large-domain structure (the diameter were around 200 μm) appeared after 6 h. This large-domain structure was retained after coking for 10 h. Moreover, the mesophase content of R2-MP-8h (a mesophase pitch prepared using R2 as raw material by thermal treatment after 8 h) was 95 % and the softening point was 243 °C. However, a mesophase pitch with coarse mosaic structure was formed because of the overreaction.

In summary, R1 leads to a mesophase pitch with medium domain structure after 8 h, while the mesophase pitch that produced from R2 had large-domain structure after 6 h. The results show that R2 can be generated into high-quality mesophase pitch under certain conditions through carbonization alone, while R1 cannot.

### Yield analysis

**Fig. 7:**
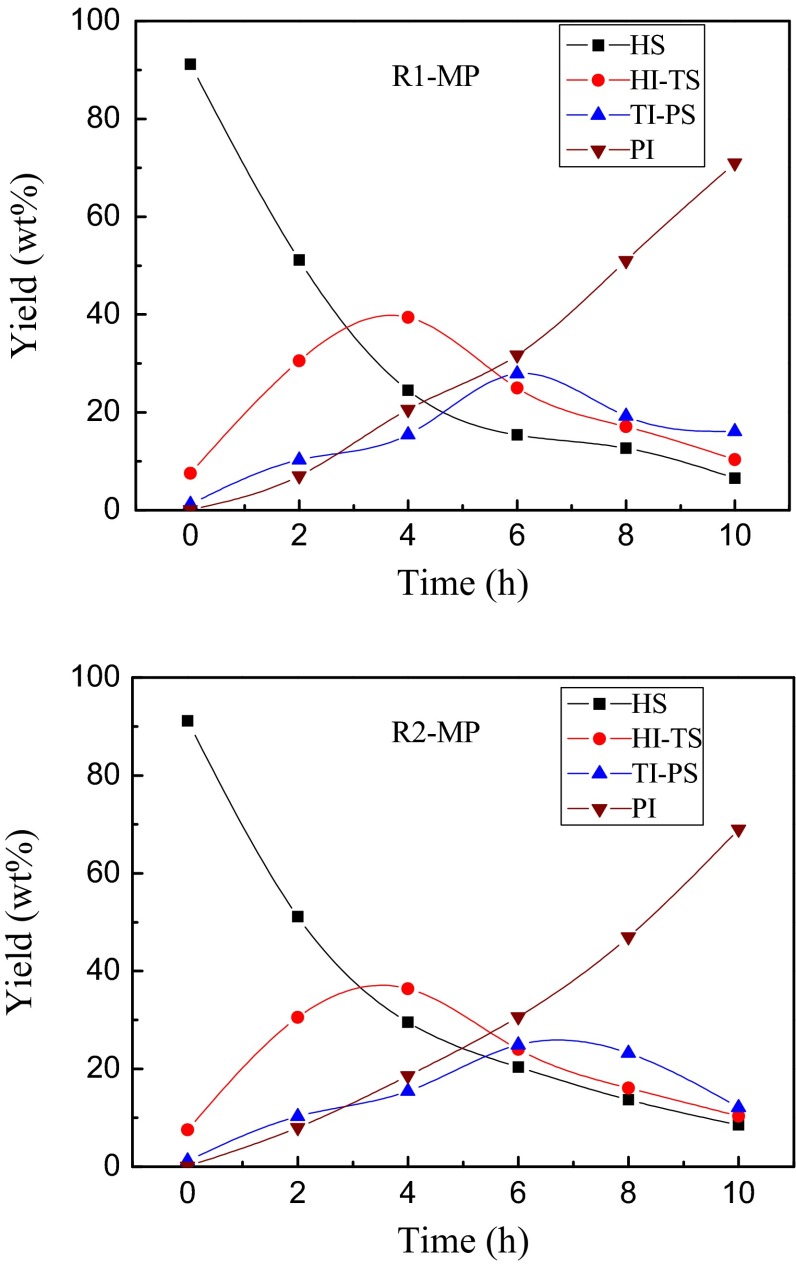
Variation of HS, HI-TS, TI-PS and PI yields (wt%) with time

Four fractions *n*-heptane-soluble fraction (HS), *n*-heptane-insoluble/toluene-soluble fraction (HI-TS), toluene-insoluble/pyridine-soluble fraction (TI-PS) and pyridine-insoluble fraction (PI) of the product are obtained by extracting with heptane, toluene and pyridine. The yields of fractions refer to the mass percentages of fractions. The yield–time curves of HS, HI-TS, TI-PS and PI in mesophase pitches are shown in Fig. 7. The changing tendencies of fraction yields in R1-MP were similar to those of R2-MP. The HS yields decreased and the PI yields increased with the proceeding of carbonizing treatment, while the HI-TS yields and TI-PS yields increased at first and then decreased. The reason was that HS-TI and TI-PS were the intermediate products and its yields changed depending on its production rate and consumption rate [22, 23]. The yield of R2-MP was lower than that of R1-MP at the end of the reaction.

During the process of thermal treatment, the HI-TS and TI-PS yields of R2-MP changed more slowly than that of R1-MP. The reason may be that the hydrogen transfer reaction took place owing to the naphthenic groups which could produce macromolecular free radicals to restrain the thermal polycondensation reaction. Thereby the system viscosity increased slowly. The lower system viscosity could help fulfill the transformation of each component, so the intermediate components were converted into the larger molecular components rapidly once they were generated. It was beneficial to generate the mesophase pitch with homogeneous molecular structure (molecular weight distribution was more concentrated).

### XRD analysis

**Fig. 8:**
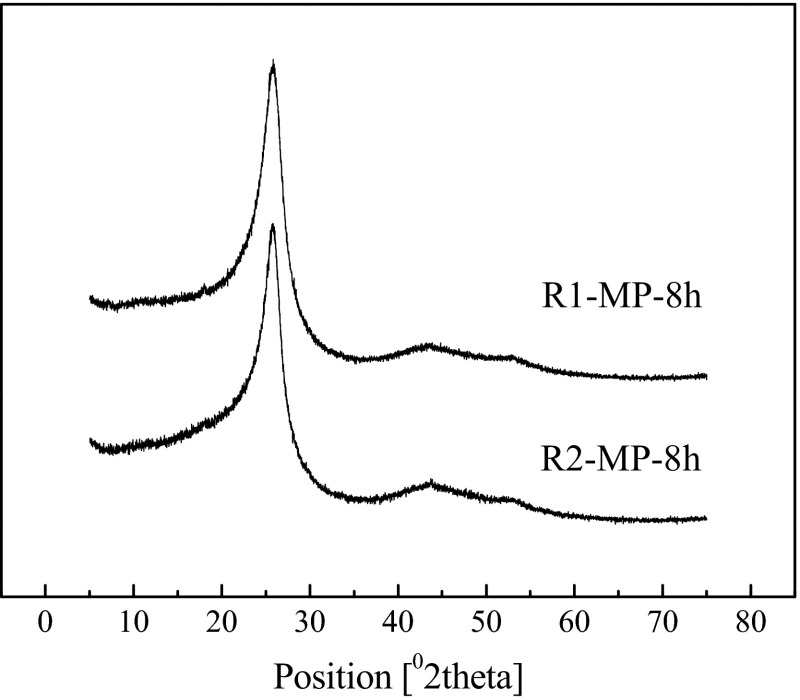
XRD of the mesophase pitches

The solubility of mesophase molecules in an isotropic matrix was determined by the distribution of mesophase molecule structures, and the movement of mesophase molecules was determined by the solubility of mesophase molecules. Thereby it affected the orientation of the aromatic layer in mesophase pitch. The X-ray spectra of R1-MP-8h and R2-MP-8h are shown in Fig. 8 and the X-ray dates are tabulated in Table 4. Two kinds of mesophase pitches exhibited strong diffraction peaks as shown in Fig. 8, which indicated that the mesophase pitches were highly crystallized. Compared with R1-MP-8h, the stack height (*L*_c_) of R2-MP-8h was higher, the interlayer spacing (*d*_002_) was lager and alignment degree (*O*_g_) was improved. As a result, the crystal structure of R2-MP-8h was more ordered.

**Table 4:** X-ray parameters of the mesophase pitches

Code	2*θ*/°	*d*_002_ (nm)	*L*_c_ (nm)	*O*_g_
R1-MP-8h	25.6686	0.3314	2.1	0.7425
R2-MP-8h	25.7437	0.3479	3.9	0.9713

Compared with R2, R1 contains more alkyl side chains, which would produce more free radicals to increase the viscosity rapidly through condensation. Then the anisotropic structure is formed when the size of molecule reaches certain level. On the other side, R2 contains more naphthenic groups. The large content of naphthenic groups leads to hydrogen transfer reaction which plays a role in easing the carbonizing reaction, so that the viscosity of the system is lowered and then the mesophase pitch molecules have enough time to rearrange. Therefore, the optical texture of R1-MP-8h presents medium domain structure, while the optical texture of R2-MP-8h was large-flow domains.

## Conclusion

The composition of raw materials played an important role on mesophase pitch formation. The raw material R2 with higher aromaticity, more naphthenic groups and less alkyl side chain was easy to form a high-quality mesophase pitch with large-domain optical texture, high mesophase content and low softening point (243 °C). During the process of thermal treatments, the intermediate fractions HI-TS and TI-PS of R2-MP changed more slowly than those of R1-MP and the thermal polycondensation reaction was restrained. The PAH of R2-MP-8h was less than that of R1-MP-8h, which was consistent with the change of aromatic indexes. Additionally, the crystal structure of R2-MP-8h was more ordered, the stack height (*L*_c_) was higher, the interlayer spacing (*d*_002_) was larger and the alignment degree (*O*_g_) was improved.
